# Development of an *E. coli* strain for cell‐free ADC manufacturing

**DOI:** 10.1002/bit.27961

**Published:** 2021-10-25

**Authors:** Dan Groff, Nina A. Carlos, Rishard Chen, Jeffrey A. Hanson, Shengwen Liang, Stephanie Armstrong, Xiaofan Li, Sihong Zhou, Alex Steiner, Trevor J. Hallam, Gang Yin

**Affiliations:** ^1^ Sutro Biopharma, Inc. San Francisco California USA; ^2^ InfinixBio Athens Ohio USA; ^3^ Present address: Rishard Chen, Geltor 1933 Davis Street San Leandro California 94577 USA

**Keywords:** antibody–drug conjugate (ADC), cell‐free protein synthesis, nonnatural amino acids

## Abstract

Recent advances in cell‐free protein synthesis have enabled the folding and assembly of full‐length antibodies at high titers with extracts from prokaryotic cells. Coupled with the facile engineering of the *Escherichia coli* translation machinery, *E. coli* based in vitro protein synthesis reactions have emerged as a leading source of IgG molecules with nonnatural amino acids incorporated at specific locations for producing homogeneous antibody–drug conjugates (ADCs). While this has been demonstrated with extract produced in batch fermentation mode, continuous extract fermentation would facilitate supplying material for large‐scale manufacturing of protein therapeutics. To accomplish this, the IgG‐folding chaperones DsbC and FkpA, and orthogonal tRNA for nonnatural amino acid production were integrated onto the chromosome with high strength constitutive promoters. This enabled co‐expression of all three factors at a consistently high level in the extract strain for the duration of a 5‐day continuous fermentation. Cell‐free protein synthesis reactions with extract produced from cells grown continuously yielded titers of IgG containing nonnatural amino acids above those from extract produced in batch fermentations. In addition, the quality of the synthesized IgGs and the potency of ADC produced with continuously fermented extract were indistinguishable from those produced with the batch extract. These experiments demonstrate that continuous fermentation of *E. coli* to produce extract for cell‐free protein synthesis is feasible and helps unlock the potential for cell‐free protein synthesis as a platform for biopharmaceutical production.

## Graphical abstract

Through chromosomal engineering, an E. coli strain was generated that will facilitate large scale manufacturing of antibody drug conjugates (ADC) with cell free protein synthesis. This E. coli strain was capable of stable coexpression of FkpA, DsbC and o‐tRNA, three factors required for cell free ADC production over a multiday continuous fermentation.

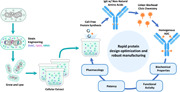

## 1 INTRODUCTION

Antibodies have desirable properties that make them particularly well suited for therapeutic applications. They are large and have an affinity for the neonatal Fc receptor which can result in serum half‐lives greater than 15 days (Booth et al., 2018). They have excellent affinities for their target, which can bind with picomolar K_D_ (equilibrium dissociation constant) (Kamat et al., 2020). Finally, they are human proteins with exquisite specificity, reducing immunogenicity and other off‐target effects (Irani et al., 2015).

Cell‐free protein synthesis (CFPS) has emerged as a powerful method for the rapid production of a variety of immunoglobulin formats including IgG, scFv, Fab, and bispecific antibodies (Yin et al., 2012; Xu et al., 2014). Previous studies inspired by the IgG folding pathway in the mammalian endoplasmic reticulum identified two key protein factors (DsbC and FkpA) for expressing immunoglobulins at high yield with in vitro protein synthesis (Groff et al., 2014). Disulfide bond isomerization is a key process in immunoglobulin maturation that allows the correct conformation of disulfide bonds to occur by allowing disulfide shuffling until the protein reaches its mature and most stable form. In prokaryotic expression systems, this process can be efficiently catalyzed by the disulfide isomerase DsbC (Yin et al., 2012; Frey et al., 2008). The other biochemical activity key for folding IgG constructs is peptidyl‐prolyl isomerase (PPI), which interconverts the thermodynamically favored *trans* proline isomer to *cis*. There is a key proline residue in the CH1 domain of the heavy chain that must be converted to *cis* before the mature IgG fold is reached (Feige et al., 2010). The periplasmic *E. coli* PPI, FkpA can facilitate this isomerization and has been shown to greatly enhance IgG folding and assembly in CFPS reactions (Groff et al., 2014).

Earlier methods for antibody conjugation utilize the reactivity of natural amino acid functional groups such as the thiol group on cysteine or the primary amine in the lysine side chain and modify a fraction of these amino acids. This leads to heterogeneity in the sites and numbers of residues modified for the IgG bioconjugate. There is a growing desire and market for antibodies with bioconjugation handles at clearly defined locations (J. Walsh et al., 2021). The production of antibodies with site‐specific conjugation handles can facilitate the production of fluorophore or metal labeled antibodies for in vivo imaging applications (Adumeau et al., 2016), precision attachment of radioisotopes for directed radiation treatment (Kitson et al., 2013), tethering of boron for boron neutron capture therapy (Wu et al., 2004), or attachment of cytotoxins to produce antibody–drug conjugates (ADCs) for oncology treatments (Kline et al., 2015). The use of site‐specific conjugation techniques also facilitates the production of homogenous antibody conjugates with desired activity and pharmacokinetic profiles (Yin et al., 2017).

There are a variety of different technologies for producing antibodies with precisely defined conjugation sites including the use of an engineered cysteine (Bhakta et al., 2013), sugar modification (Pergolizzi et al., 2016), or introduction of small tags for enzyme‐mediated conjugation including transglutaminase or aldehyde tags (Falck & Müller, 2018). Nonnatural amino acid (nnAA) mutagenesis offers a particularly advantageous method for site‐specific bioconjugation because it can utilize biorthogonal chemistries, eliminating any off‐target conjugation with naturally occurring amino acids (Axup et al., 2012). This technique requires mutation of only a single amino acid residue, allowing the conjugation site to be placed at any residue in the protein which does not impact folding, effector functions, or substrate interactions while preserving biological activity that could be affected by the introduction of a larger tag.

nnAA mutagenesis utilizes an amber suppressor orthogonal tRNA‐AAtRNA (aminoacyl tRNA) synthetase system which incorporates a nnAA co‐translationally in response to the TAG “amber” stop codon. In mammalian cell lines, clinical scale antibody production has been demonstrated with this system using stable cell lines with chromosomally integrated tRNA and synthetase (Axup et al., 2012). However, nnAA incorporation at TAG codons competes with translational termination. This limits the efficiency of nnAA incorporation, precludes nnAA mutagenesis at difficult‐to‐suppress sites, and typically restricts the number of nnAA incorporated to one per protein chain (Axup et al., 2012). As an alternative to mammalian cell production, CFPS of nnAA containing IgGs offers some important advantages. First, the open nature of CFPS allows components of the nnAA incorporation machinery to be directly added to the reaction. This is particularly important for the nnAA itself which would otherwise need to cross the cell membrane in a cell‐based expression system (Zimmerman et al., 2014). The translational machinery of *E. coli* is also easier to modify than mammalian cells, which facilitates knockout or engineering of factors responsible for translational termination, enabling the incorporation of multiple nnAAs and incorporation at challenging sites (Johnson et al., 2011; Yin et al., 2017). Finally, in CFPS, growth is decoupled from protein synthesis and proteins essential for growth such as RF1, which terminates translation at TAG codons, can be removed through proteolysis before in vitro protein synthesis. IgG expression in extract lacking RF1 allows efficient production of IgGs with up to 8 nnAAs (Yin et al., 2017).

**Scheme 1:**
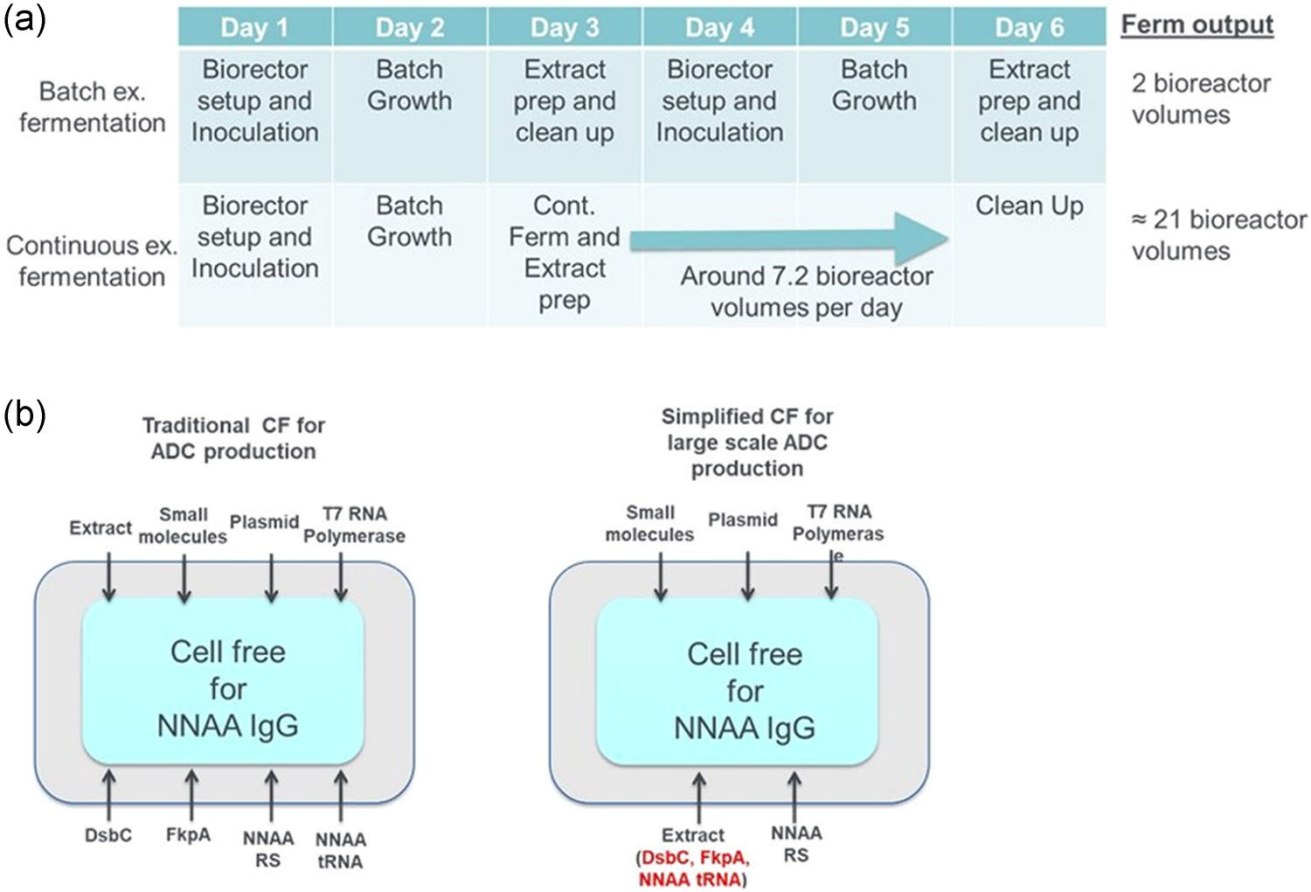
Process improvements to facilitate ADC production with cell‐free (CF) protein synthesis. (a) Process flow and bioreactor output for batch and continuous extract strain fermentations. (b) Simplified CF setup when extract strain coexpresses chaperones and o‐tRNA for nnAA incorporation. ADC, antibody–drug conjugate; nnAA, nonnatural amino acid

One of the most important challenges with CFPS of nnAA IgGs at larger scales is access to sufficient quantities of extract. Extract production conventionally requires batch or fed‐batch cell fermentations where cells are grown to a high OD in a bioreactor and the biomass is harvested in a single pass. Batch extract production is highly successful at smaller scales, however, it becomes limiting at the scales needed for commercial production of therapeutic antibodies in a CFPS system. One way to address this challenge is to grow the extract strain chemostatically at high cell density and rates of division. This mode of fermentation has the potential to increase the *E. coli* cell extract output for a bioreactor 20 times (Kopp et al., 2019 and Scheme 1a) While in continuous fermentation mode, cells are grown much longer than typical batch or fed‐batch fermentation and go through many more rounds of division. This necessitates strains with high genetic stability which is complicated by the fact that our previously engineered extract strains overexpress chaperones from a plasmid with antibiotic selection for plasmid maintenance (Groff et al., 2014). Antibiotic selection is less effective at high cell density and continuous fermentation would generate a large volume of antibiotic contaminated waste (Velur Selvamani et al., 2014). To address these issues, an auxotrophic selection scheme was devised, based on glutamine auxotrophy, that enabled the retention of a plasmid for at least a week of continuous fermentation. However, over the course of this fermentation, instability within the expression cassette resulted in decreasing levels of FkpA. This problem was solved by integrating expression cassettes for both chaperones onto the chromosome, resulting in stable expression of both DsbC and FkpA at g/L quantities in the extract.

To facilitate site‐specific ADC production with nnAAs it would also be desirable to produce the amber suppressor tRNA during extract fermentation. Our previous chaperone studies suggested that stable, high‐level expression of the amber suppressor tRNA should be achievable with genes transcribed from the chromosome (Groff et al., 2014). Initial studies with a synthetic orthogonal tRNA (*o*‐tRNA) operon demonstrated that tRNA activity correlated very well with promoter strength. Unfortunately, it also became clear that even with the strongest promoter tested, a single integrated *o*‐tRNA operon could not achieve tRNA levels sufficient for maximal amber suppression during cell‐free nnAA‐IgG synthesis. This is a common challenge in moving from a multicopy plasmid to a single copy chromosomal integrant. One elegant solution for dealing with this challenge called Chemically Induced Chromosomal Evolution utilizes recA to form up to 40 concatemerized gene or pathway duplications (Tyo et al., 2009). Implementing this approach offered a 10‐fold stability enhancement relative to plasmid expression of the same pathway. However, over time the copy numbers were reduced by homologous recombination. From initial inoculum growth and week‐long continuous fermentation, the extract strain may be growing for as long as 9 days, therefore necessitating even greater stability. This was achieved through the integration of the *o*‐tRNA operon onto the chromosome at multiple defined sites which allowed us to spatially separate these genes and precisely control and optimize *o*‐tRNA levels within the extract strain. This also demonstrates the utility in expressing factors for CFPS from the chromosome. Further improvements can be prototyped through plasmid‐based overexpression before genes are moved onto the chromosome in an iterative process.

The final ADC‐extract strain was capable of constitutive expression of the chaperones DsbC and FkpA and *o*‐tRNA from genes integrated into the chromosome. All three were expressed at quantities sufficient for high‐level nnAA‐IgG synthesis in CFPS, simplifying the reaction setup and eliminating one reagent fermentation and lysate production needed for ADC production (Scheme 1b). Extracts produced from these cells showed consistent chaperone levels, IgG synthesis, and nnAA incorporation on the first and final days of continuous fermentation, indicating that these chromosomally integrated genes were stable. In addition, extract produced from continuously grown cells had cell‐free performance comparable to batch‐produced extract demonstrating that continuously growing cells for extract production is a viable route for increasing yields of cell‐free extract—a key requirement for enabling commercial production of biotherapeutics by CFPS.

## 2 MATERIALS AND METHODS

### 2.1 General molecular biology

*E. coli* DH10b [F^−^ *endA1 recA1 galE15 galK16 nupG rpsL* Δ*lacX74* ϕ80d*lacZ*ΔM15 *araD139* Δ(*ara,leu*)*7697 mcrA* Δ(*mrr‐hsdRMS‐mcrBC*) λ^−^] was used for plasmid construction and propagation. Cells were maintained in Luria broth (LB) or Terrific Broth (TB). For plasmid selection, media was supplemented with 50 μg/ml kanamycin and 100 μg/ml carbenicillin where appropriate. Complex media and antibiotics were purchased from Teknova. All plasmid construction was performed using the Choo‐Choo homology cloning kit from McLab, unless noted otherwise. All polymerase chain reactions (PCRs) were performed using Phusion DNA polymerase from New England Biolabs. Strains utilized in this study can be seen in Table 1.

**Table 1:** List of strains used in this study

Strain	Modification	Reference
SBHS016	RF1 N296K/L297R/L298R	Yin et al. (2017)
SBEZ023	SBHS016 + pJ201 pAzPhe tRNA	Yin et al. (2017)
SBDG098	SBHS016 Δ*glnA*	This paper
SBDG099	SBHS016 Δ*cysE*	This paper
SBDG100	SBHS016 Δ*argA*	This paper
SBMT095	*galK*::Pc0‐2XDsbC	Groff et al. (2014)
SBDG108	SBMT95 + pACYC‐Pc0 2xFkpA	Groff et al. (2014)
SBDG159	SBMT95 Δ*glnA*	This paper
SBDG161	SBDG159 + pACYC‐Pc0 2xFkpA‐CP42 *glnA*	This paper
SBDG112	SBHS016 xylA::Pc0 2XDsbC	This paper
SBDG150	SBDG112 galK::Pc0‐2XFkpA	This paper
SBDG160	SBDG150 ΔglnA	This paper
SBDG224	SBDG160 + pACYC‐Pc0 pAF tRNA‐CP42 *glnA*	This paper
SBDG265	SBDG150 *araB*::pAzPhe‐tRNA_proK promoter	This paper
SBDG266	SBDG150 *araB*::pAzPhe‐tRNA_Sutro promoter PL6	This paper
SBDG267	SBDG150 *araB*::pAzPhe‐tRNA_MTL promoter with mutation	This paper
SBDG268	SBDG150 *araB*::pAzPhe‐tRNA_MTL promoter	This paper
SBDG275	SBDG266 *sdaB* scar::MTL prom‐pAzPhe tRNA	This paper
SBDG276	SBDG268 *sdaB* scar::MTL prom‐pAzPhe tRNA	This paper
SBDG286	SBDG276 *tonA* scar:: MTL promoter pAzPhe tRNA‐pla3 tRNA	This paper
SBDG287	SBDG276 *tonA* scar:: MTL promoter pAzPhe tRNA	This paper
SBDG299	SBDG287 *gshA* scar::MTL promoter‐pAzPhe tRNA	This paper
SBDG300	SBDG287 *gshA* scar::MTL promoter‐pAzPhe tRNA_pla3‐tRNA	This paper
SBDG301	SBDG286 *gshA* scar::MTL promoter‐pAzPhe tRNA	This paper
SBDG302	SBDG286 *gshA* scar::MTL promoter‐pAzPhe tRNA_pla3‐tRNA	This paper
SBDG310	SBDG287 *tnaA* scar::MTL promoter‐pAzPhe tRNA	This paper
SBDG311	SBDG287 *tnaA* scar::MTL promoter‐pAzPhe tRNA_pla3‐tRNA	This paper
SBDG312	SBDG286 *tnaA* scar::MTL promoter‐pAzPhe tRNA	This paper
SBDG313	SBDG286 *tnaA* scar::MTL promoter‐pAzPhe tRNA_pla3‐tRNA	This paper
SBDG316	SBDG299 *tnaA* scar::MTL promoter‐pAzPhe‐tRNA	This paper
SBDG317	SBDG299 *tnaA* scar::MTL promoter‐pAzPhe_pla3‐tRNA	This paper
SBDG318	SBDG302 *tnaA* scar::MTL promoterp‐AzPhe‐tRNA	This paper
SBDG319	SBDG302 *tnaA* scar::MTL promoter‐pAzPhe_pla3‐tRNA	This paper

### 2.2 Auxotrophic selection

Starting strains were made auxotrophic for amino acids by disrupting *glnA, cysE*, or *argA*, which is required for the biosynthesis of glutamine, cysteine, or arginine respectively from the genome of the cell‐free optimized *E. coli* host. Gene knockouts were performed using homologous recombination with a selection marker as described previously (Datsenko & Wanner, 2000).

**Figure 1:**
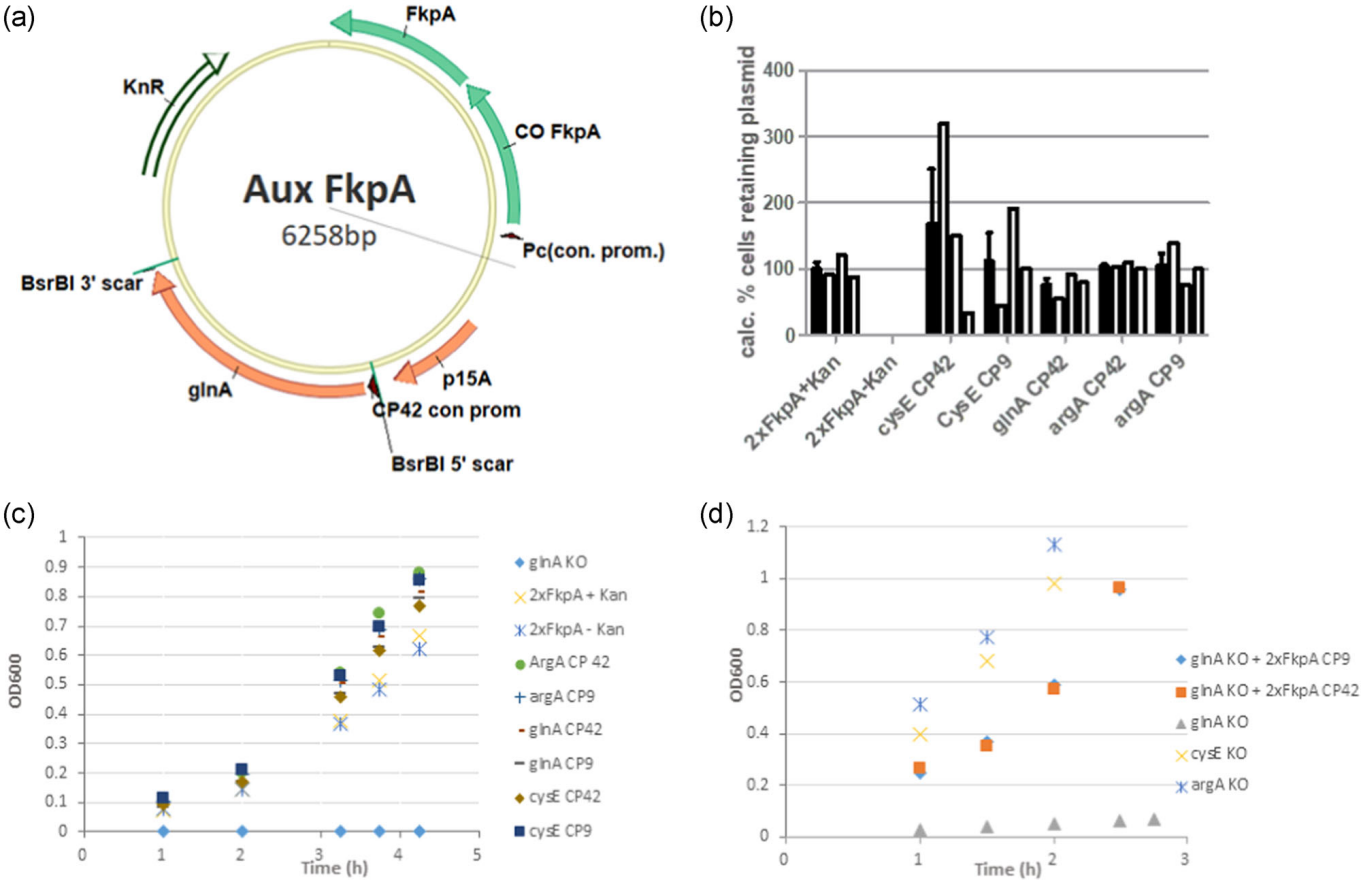
Auxotrophic selection for continuous fermentation. (a) Example of a vector for FkpA overexpression in continuous fermentation in a glutamine auxotrophic strain background. (b) Plasmid retention with antibiotic and auxotrophic selection as measured by the ratio of colonies on media with or without kanamycin; 2xFkpA uses traditional antibiotic selection for plasmid maintenance. White bars show the average of three biological replicates shown in black. Growth of strains in (c) M9 minimal media; (d) the complex media terrific broth. Both graphs show data from a single representative sample

The complementing plasmids used in this study are based upon the medium copy ACYC origin of replication. This auxotrophic plasmid system has been used for the expression of FkpA and an *o*‐tRNA for nnAA incorporation. An example of this type of plasmid is shown in Figure 1a. For cloning this variant of restoring plasmid, miniprepped pACYC‐PC plasmid was cut at a single BsrBI site. *glnA, cysE*, or *argA* was amplified out of WT *E. coli* strain SBJY001 with PCR primers that had homology to sequence 3ʹ and 5ʹ of the BsrBI restriction site. These PCR primers also introduced a strong constitutive promoter, CP9, or medium strength constitutive promoter, CP42, upstream of each gene, responsible for its transcription. This fragment was then cloned into pACYC‐PC using choo‐choo cloning (McLab).

To prepare strains with auxotrophic selection, mutants SBDG098, SBDG099, SBDG100 lacking *glnA, cysE*, or *argA* respectively, were made electrocompetent and then transformed with a complementing plasmid. The plasmid could be maintained in media with kanamycin or media lacking the specific amino acid.

Plasmid retention was measured with a plate assay. Stationary phase cultures were diluted 1 × 10^7^ into LB broth and plated onto LB agar plates with and without kanamycin. The retention rate was calculated by dividing the number of colonies on the kan plates by the number of colonies that grew without antibiotics.

Growth assays were performed by picking colonies off LB‐Agar plates, inoculating in MOPS minimal media with 0.1% glucose, and incubating at 37°C degrees overnight in a shaking incubator at 250 RPM. Samples that grew to an OD595 > 0.25 were considered positive for growth.

### 2.3 Integration of FkpA and tRNA strain expression cassettes

A bacterial strain SBDG112 derived from SBHS016 was used as the initial source strain. The FkpA and tRNA expression strains were constructed using previously described methods (Groff et al., 2012). To insert the tandem copies of FkpA into strain SBDG112, the FkpA integration cassette, containing two copies of FkpA genes with a Pc0 promoter and kanamycin selection marker flanked by *galK* homology arms, was constructed by PCR, and was then electroporated into SBDG112 expressing lambda red recombinase and selected on LB with 50 μg/ml kanamycin plates (Datsenko & Wanner, 2000).

Cre recombinase expression from the pJW168 plasmid was then used to remove the kanamycin resistance marker. The removal of the kanamycin gene was confirmed by colony PCR, and the integrated tandem FkpA gene sequence was confirmed by DNA sequencing.

The resulting FkpA expressing strain SBDG150 was used for tRNA integrations. The tRNA integration cassettes were constructed by PCR to add a constitutive promoter and contain the tRNA sequences and kanamycin gene flanked by the *araB* homology arm sequences. tRNA integration cassettes contained either the pAzPhe tRNA or the pAzPhe tRNA followed by the pla3 tRNA separated by the natural tRNA linker between *hisR* and *leuT* tRNAs in the *E. coli* genome (Guo et al., 2009). Both of these o‐tRNAs are recognized by nnAA derivatives of the mutant *Methanococcus jannaschii* tyrosyl aminoacyl tRNA synthetase used in this study, but this prevents duplicating the same gene twice, which facilitates molecular biology and stability in the strain. The tRNA integration cassette was then electroporated into SBDG150 strain expressing lambda red recombinase and selected on LB plates with 50 μg/ml kanamycin. A similar method was used to insert the tRNA sequence into other selected target genomic sites. For the integration into the *araB* site, the entire gene was replaced. For the *sdaB, tonA, gshA*, and *tnaA* integrations, Frt scar sites from previous gene deletions were used as the integration site. The kanamycin resistance marker was removed using the method described above. The sequence of integrated tRNA sequence and promoter was confirmed by DNA sequencing.

### 2.4 Cell banking

A single colony is picked to inoculate 25 mL LB media in a Corning 125 ml Erlenmeyer flask incubated in 37°C, 250 rpm shaker. During the exponential phase (OD_595_ ~ 1), 6.25 ml of 80% glycerol is added to the flask culture and 2 ml aliquots of the cell stock are flash‐frozen in liquid nitrogen.

### 2.5 Cell extract prepared from 200 ml chemostat fermentations

Cell extracts were prepared using derivatives of Sutro extract strain SBHS016, which has a protease‐sensitive RF1 that is degraded during extract production. Strains were cultured to an OD_595_ approximately 50, grown using glucose and an amino acid fed‐batch continuous fermentation (maximum growth rate µ of 0.3 1/h). Fermentations were performed in a 250 ml Eppendorf Dasbox bioreactor equipped with pH, DO, and temperature control.

### 2.6 Cell growth and harvest

For continuous fermentation, a primary seed flask containing 10 ml 1 × 80/80 media from Table 2 was inoculated with a 2‐ml cell bank in a Corning 125 ml Erlenmeyer flask incubated at 37°C, with 250 rpm shaking. Continuous fermentation occurred in an Eppendorf DASbox bioreactor with 200 ml 1 × 80/80 media. Growth was initiated by inoculation with a 1‐ml seed culture during the exponential phase (A_595_ ~ 1 OD). Cells were grown in the bioreactor until initial glucose was depleted. Then an exponential feed and harvest rate of 0.3 1/h was initiated to prevent excess glucose accumulation. Cells were maintained in continuous fermentation at this growth rate with an OD_595_ ~ 50. The temperature was controlled at 37°C, dissolved oxygen was maintained at 30%, and pH = 7.2 was maintained through the addition of 1 M ammonium hydroxide. The volume of culture was maintained at 200 ml using a level probe.

**Table 2:** Media for continuous fermentation

Reagent	Reaction Concentration	Unit
10× step 1		
Potassium phosphate monobasic	30	g/L
Potassium phosphate dibasic	45.8	g/L
Ammonium sulfate	15	g/L
Sodium citrate dihydrate	12.75	g/L
Potassium chloride	10	g/L
Trace metals	3	ml/L
10× step 2		
Potassium hydroxide	96.5	g/L
l‐Asparagine·H2O	1.8545	g/L
Glycine	3.3772	g/L
l‐Histidine	0.5803	g/L
l‐Isoleucine	1.6638	g/L
l‐Leucine	1.9327	g/L
l‐Lysine·HCl	2.3865	g/L
l‐Methionine	0.8874	g/L
l‐Phenylalanine	0.9065	g/L
l‐Proline	2.2071	g/L
l‐Threonine	2.2765	g/L
l‐Tryptophan	0.5652	g/L
l‐Tyrosine	0.9772	g/L
l‐Valine	1.0643	g/L
Betaine·HCl	2.0084	g/L
Choline chloride	0.028638	g/L
Nicotinic acid (niacin)	0.02512	g/L
*p*‐aminobenzoic acid	0.025600	g/L
Calcium pantothenate	0.009397	g/L
Pyridoxine (B6) (HCl)	0.001471	g/L
Riboflavin (B2)	0.003886	g/L
Thiamine (B1) (HCl)	0.01766	g/L
Biotin (vitamin H)	0.11418	mg/L
Cyanocobalamin (vitamin B12)	0.00858	mg/L
Folic acid	0.06534	mg/L
1×80/80 media		
10× step 1	100	ml/L
10× step 2	100	ml/L
50% glucose	10	ml/L
Sulfuric acid	3.4	ml/L
1.31 M magnesium sulfate heptahydrate	2.4	ml/L
50% antifoam 204	0.909	ml/L

### 2.7 Biomass processing

Cells were pelleted by centrifugation using a Beckman Coulter J‐26 XPI floor centrifuge at 10,000 rpm for 15 min at 4°C. Pellets were resuspended in five times S30 buffer (Zawada, 2012) then pelleted again in the floor centrifuge at the same conditions. Then, pellets were resuspended in 2 volumes 1×S30 buffer and lyzed in an EmulsiFlex‐C5 Avestin homogenizer at 14,000 psi with a single pass. Lysate was held between 0°C and 4°C after lysis. To remove the insoluble fraction, the lyzed extract was centrifuged two more times at 17,000 rpm for 30 min at 4°C. The extract was aliquoted into 50 ml falcon tubes and flash‐frozen in liquid nitrogen to be stored at −80°C. For activation, extract was thawed and placed in a 40°C water bath for 40 min and then chilled on wet ice. Post activation, DsbC, and FkpA levels were assessed with a coating ELISA as previously described (Groff et al., 2014).

### 2.8 Cell‐free protein synthesis at 1 ml scale

An aCD74 IgG was expressed in a CFPS reaction as previously described with the following modifications (Abrahams et al., 2018). Cell‐free extract was thawed and treated with 75 μM iodoacetamide for 30 min at ambient temperature (20°C) then added to premix containing all the components including the IgG heavy and light chain DNA. A 1 ml cell free reaction containing 37.5% (v/v) iodoacetamide treated extract, HC and LC plasmids, NMPs, amino acids, and other small molecules previously described (Zawada et al., 2011) and was run overnight for 16–18 h in a flowerplate (m2p‐labs) at 29°C with 650 rpm agitation on a thermomixer R (Eppendorf). Proteins produced were purified with PhyNexus Phytip ProPlus columns and quantified using A280 measurements. For expressing aCD74 Phe404pAMF, the reaction was supplemented with 1.2% lysate from cells overexpressing the pAMF aminoacyl tRNA synthetase (Zimmerman et al., 2014), and 2 mM *p*‐azidomethyl phenylalanine·HCL (pAMF).

### 2.9 Bioreactor expression, purification, and characterization of aCD74 IgG

CFPS IgG production was carried out in a stirred tank bioreactor (DASgip, Eppendorf) as described previously. Reactions were performed with a 0.25‐L volume at 29°C. pH and DO were controlled at 7.2% and 20%, respectively for 14 h followed by a shift to pH 8.0% and 80% DO. The CFPS reaction was harvested by centrifugation and the supernatant was captured with Protein A affinity resin (MabSelect Sure) and eluted with 100 mM Glycine pH 3.2. The neutralized ProA pool was concentrated and polished by preparative SEC (Superdex 200) in phosphate‐buffered saline (PBS). Product quality was accessed by analytical SEC (Zenix‐C SEC‐300, 3 μM, 7.8 × 300 mm, Sepax Technologies) with a mobile phase of 50 mM sodium phosphate, 140 mM NaCl, 5% isopropanol, pH 6.5. Two reactions were set up with extract produced continuously, and one reaction was set up using extract produced with a batch fermentation for comparison. Titers of all three reactions were measured as described for 1 ml scale in duplicate for each bioreactor.

### 2.10 ADC conjugation and characterization

Polished aCD74 IgG was diluted to 1 mg/ml in PBS and incubated at room temperature overnight with a three‐fold molar excess (40 μM) of DBCO‐maytansine drug‐linker. Excess un‐reacted drug‐linker was removed by desalting spin colum (Zeba spin desalting plate, 7 K MWCO, Thermo Fisher Scientific) equilibrated with PBS. Product quality was accessed ay analytical SEC and the Drug Antibody Ratio (DAR) was determined by reduced matrix‐assisted laser desorption ionization time‐of‐flight mass spectrometry (MALDI‐TOF). Briefly, protein was reduced with 10 mM TCEP for 20 min at 37°C, diluted 1:1 with S‐DHB matrix (50 mg/ml in 30% acetonitrile:70% water w 0.1% TFA) and dried on a groud‐steel MALDI target. Spectra were acquired on a Bruker autoflex speed MALDI‐TOF MS and DAR was calculated by peak height.

### 2.11 Cell lines and cell culture conditions

Mino, SU‐DHL‐6, and CHO‐k cells were purchased from ATCC (American Type Culture Collection), OPM2 cells were purchased from The Leibniz Institute DSMZ (German Collection of Microorganisms and Cell Cultures GmbH). CHO‐humanCD74 cell line was generated by transfecting CHO‐k cells with a plasmid containing human CD74 cDNA sequence and selecting for the highest stable expression of humanCD74 on the cell surface.

Mino, SU‐DHL‐6, and OPM2 cells were maintained in RPMI, high glucose (Corning) supplemented with 20% heat‐inactivated fetal bovine serum (Thermo Fisher Scientific), 2 mM glutamax (Thermo Fisher Scientific), and 1× Penicillin/streptomycin (Corning). CHO‐humanCD74 and CHO‐k cells were maintained in RPMI, high glucose (Corning) supplemented with 10% heat‐inactivated fetal bovine serum (Thermo Fisher Scientific), 2 mM glutamax (Thermo Fisher Scientific), and 1× Penicillin/streptomycin (Corning).

### 2.12 Cell‐binding assay

Cells were harvested and re‐suspended in FACS buffer (DPBS buffer supplemented with 1% bovine serum albumin). A total of 200,000 cells per well were incubated on ice with serial dilutions of IgG for 60 min. Cells were washed twice with ice‐cold FACS buffer and then incubated with 5 μg/ml Alexa 647 labeled donkey anti‐human Fc antibody (Jackson ImmunoResearch) on ice for another 60 min. Unstained cells and cells stained with secondary antibody alone were used as controls. Samples were then washed twice using FACS buffer and analyzed using a BD FACS Canto system. Geometric mean fluorescence intensities (MFI) were fitted using nonlinear regression analysis with one site‐specific binding equation on GraphPad Prism. Data was expressed as MFI versus antibody concentration in nanomolar (nM).

### 2.13 ADC cell killing assay

Cytotoxicity effects of the ADCs were measured with a cell proliferation assay. A total of 12,500 cells in a volume of 25 μl were seeded in a 384‐well flat bottom white polystyrene plate on the day of assay. Free linker‐warheads and ADCs were formulated at two times of starting concentration (100 nM) in cell culture medium and filtered through MultiScreen HTS 96‐Well Filter Plates (Millipore). Filter sterilized samples were serially diluted (1:3) under sterile conditions and added onto cells in quadruplicates. Plates were cultured at 37°C in a CO_2_ incubator for 72 h. For cell viability measurement, 30 μl of Cell Titer‐Glo® reagent (Promega Corp) were added into each well, and plates processed as per product instructions. Relative luminescence was measured on an ENVISION® plate reader (Perkin‐Elmer). Relative luminescence readings were converted to % viability using untreated cells as controls. Data was fitted with nonlinear regression analysis, using log(inhibitor) versus response, variable slope, 4‐parameter fit equation using GraphPad Prism. Data was expressed as % relative cell viability versus dose of free linker‐warhead or ADC in nM with error bars indicating the standard deviation (*SD*) of the quadruplicates.

## 3 RESULTS

### 3.1 Auxotrophic selection

Overexpression of the chaperones DsbC and FkpA in extract is required for the folding and assembly of IgGs during CFPS. It was previously demonstrated that more than 1 g/L IgG titers were achievable with an extract strain in which DsbC was overexpressed from the chromosome and FkpA was overexpressed from a medium copy plasmid with a p15A origin (Groff et al., 2014). Though we were initially interested in adapting this system for compatibility with continuous fermentation, antibiotic selection is not desirable in continuous fermentation because the high cell density reduces antibiotic concentration and decreases selection pressure for plasmid maintenance (Kopp et al., 2019). In addition, the large culture volumes associated with continuous fermentation produce a large volume of antibiotic‐contaminated spent media. To address these issues, we have opted to maintain the plasmids in our continuous fermentation strains with auxotrophic selection by knocking out an essential gene required for amino acid biosynthesis and rescuing with gene expression from a plasmid (Figure 1a).

The media for continuous fermentation is a defined media with mineral salts, glucose, vitamins, and 13 amino acids. That leaves 7 amino acids that could potentially be targeted by auxotrophic selection. Of these remaining amino acids, we identified three whose biosynthesis could be disrupted by deletion of a single gene. These three genes were *cysE* for cysteine biosynthesis, *glnA* for glutamine biosynthesis, and *argA* for arginine biosynthesis. Initially, we verified that mutants with these single‐gene knockouts were unable to grow in our continuous media (data not shown). To test the ability of each auxotrophic system to work for plasmid selection, strains with disrupted amino acid biosynthesis pathways were transformed with the complementing plasmid and grown in minimal media. In Figure 1b, the auxotrophic selection system maintained the plasmid in nearly 100% of cells for all three amino acid systems, comparable to antibiotic selection with both promoters, (CP9 and CP42) indicating the selection was robust with respect to GlnA expression levels. In the absence of selection, kanamycin‐resistant cells were not detectable indicating the plasmid was lost from these cells. Previous research indicates plasmid selected by auxotrophy will also be unstable if they don't confer a growth advantage (Dong et al., 2010). Figure 1c and S2 shows each of the strains with restored amino acid biosynthesis had growth rates similar to the prototrophic parental strain in minimal media, while the untransformed strains did not grow (only the Gln auxotroph is shown for simplicity). We were also interested in testing whether this auxotrophic selection system would function in complex media which would contain all 20 amino acids. Figure 1d shows the growth of the untransformed auxotrophic *cysE, argA*, and *glnA* mutants. The *cysE* and *argA* mutants grew at about the r same rate as the transformed glnA strain with an intact glutamine biosynthesis pathway in these conditions, while the *glnA* mutant grew significantly slower in complex media. A faster growth rate was restored after transformation with the complementing plasmid. This result suggests that *glnA* and the glutamine biosynthesis pathway may have other important roles in the cell beyond the synthesis of glutamine and this system confers selectivity even in glutamine‐containing media. Because of its more robust selectivity, the glnA system was used in media without glutamine for future work requiring auxotrophic selection.

### 3.2 Continuous fermentation with FkpA expressed from plasmid

**Figure 2:**
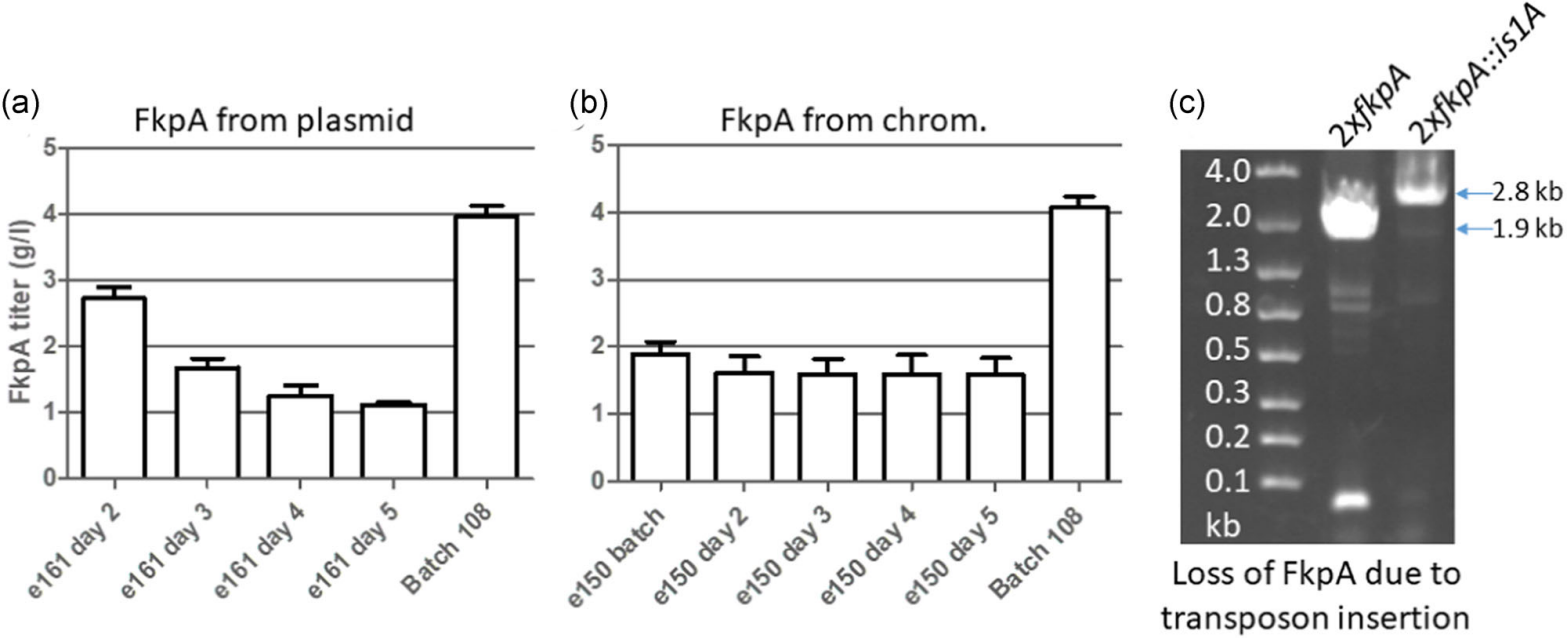
Continuous fermentation with plasmid or genomic FkpA overexpression. The control strain 108 is a batch extract strain with DsbC expression from the chromosome and FkpA expression from a plasmid with antibiotic selection (Groff et al., 2014). (a) FkpA overexpression from a plasmid (b) FkpA overexpression from the chromosome. In (a) and (b) extract samples were made at the indicated timepoints and FkpA levels were measured with a coating ELISA using polyclonal FkpA antisera. (c) Shows polymerase chain reaction (PCR) amplification of the FkpA expression cassette from an FkpA overexpressing strain on the left. The right lane shows PCR amplification of a mutant FkpA expression cassette with inserted transposon in an FkpA nonexpressing cell from fermentation in (a) after 5 days. The transposon increases the size of the expression cassette and PCR amplicon. ELISA, enzyme‐linked immunosorbent assay

A plasmid was created to adapt our batch extract strain, SBDG108, for continuous fermentation. An FkpA expression plasmid with auxotrophic selection was cloned with 2 copies of FkpA behind the Pc0 promoter and the *glnA* gene with a medium strength constitutive promoter, CP42 (Figure 1a). The two copies of FkpA were encoded with different codon utilization to suppress homologous recombination between the two *fkpA* genes. This plasmid was transformed into a strain that overexpressed DsbC from the chromosome that had *glnA* deleted to produce strain SBDG161. After transformation, this strain demonstrated normal growth rates in media lacking glutamine. This strain was able to grow in continuous fermentation at an OD_600_ around 50 with a dilution rate µ = 0.3/h. This auxotrophic selection strain exhibited stable growth under these conditions for at least 5 days. After 5 days of continuous fermentation, the plasmid was also retained in nearly all of the cells in the continuous culture, as determined by plating on kanamycin (Figure S1). The auxotrophically selected plasmid was responsible for FkpA overexpression in strain SBDG161. The chaperone levels in the extract produced from cells grown in continuous fermentation were analyzed for DsbC and FkpA with PAGE and ELISA. From the ELISA measurements, it appears that the levels of FkpA are decreasing over the course of a 5‐day continuous fermentation (Figure 2a). DsbC is expressed from the same chromosomal operon in all strains used in these studies. These strains had similar expression of DsbC with stable production during the fermentation (Figures S3 and S4). PAGE gels run with extract from this continuous fermentation confirm this observation (Figure S4). Extract from this strain has approximately 4 g/L FkpA at the start of continuous fermentation, but extract made on Day 5 only contains 1.4 g/L FkpA. To try and understand this loss of FkpA production, individual cells from the Day 5 chemostat were isolated and assayed for FkpA production. A distribution of cells was observed in the culture that had been growing for 5 days. There were cells with expected high FkpA production and cells with no FkpA overexpression (Figure S5). PCR amplification of the FkpA expression cassette from the plasmid of the FkpA non‐expressors indicated that a mutation had occurred (Figure 2c). Sequencing the mutant plasmid confirmed the presence of an 867 nucleotide *is1A* transposable element inserted into the promoter for FkpA expression. While this strain was capable of producing high levels of FkpA for a short period of time, transposon mutants overtook the FkpA overexpressing cells. After 5 days of continuous fermentation, the transposon mutants represented 75% of the cells in the culture (Figure S5). Plasmid based expression is a useful tool for screening chaperones and other factors before integration onto the chromosome. However, we think chromosomal integration appears desirable for chaperones expressed in extract as plasmid based expression was unstable because of transposon mutations.

### 3.3 FkpA overexpression from the chromosome

DsbC is expressed from the chromosome in strain SBDG161. We observed stable DsbC levels during continuous fermentation over a 5‐day period (Figures S3 and S4). We wanted to see if that same strategy could be used for stable FkpA expression during continuous fermentation. To test this strategy, cell line SBDG150 was made by integrating the FkpA overexpression cassette described above into the *galK* locus in a strain with a DsbC overexpression cassette in the *xylA* locus. This strain was grown in a continuous manner and extract was prepared from continuously grown cells at the indicated time points (Figure 2b). At the earliest time point, extract made from SBDG161 had higher FkpA levels, which is not surprising because FkpA in these cells is expressed from a p15A plasmid which should have a copy number 10–15 times higher than the chromosome. However, during the course of the continuous fermentation, the FkpA levels for SBDG150 were relatively stable at around 2 g/L or about 50% of the FkpA titers from the plasmid‐based FkpA production at its highest time point. After 4 days of continuous fermentation, extract made from these cells had higher FkpA concentrations than plasmid expressed FkpA in SBDG161. Overexpressing both chaperones off the chromosome allowed continuous cell growth for extract production and stable, high‐level production (>1 g/L) of two different chaperones for at least 5 days.

### 3.4 Chromosomal expression of *o*‐tRNA

**Figure 3:**
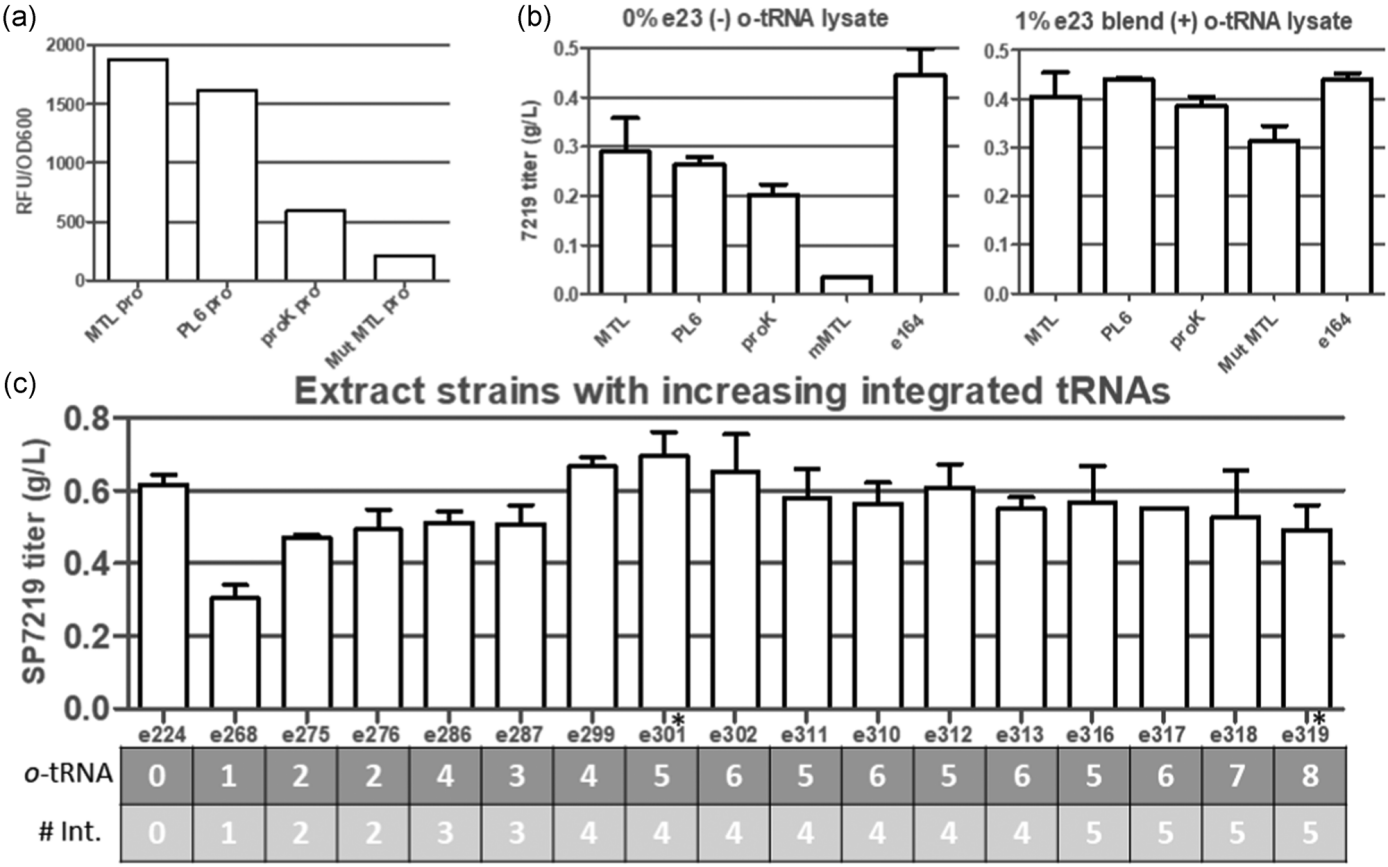
Transcription of *o*‐tRNA from chromosome. (a) Promoter library strength was measured by plasmid‐based GFP expression from a single representative sample (b) nnAA‐IgG titers from extracts with a single integrated tRNA cassette with various promoters. On the left panel, all amber suppressor tRNA is supplied by the extract. The right panel shows reactions supplemented with tRNA using an *o*‐tRNA reagent lysate e23. E164 is a batch extract with *o*‐tRNA transcription from a plasmid. (c) Optimization of the number of chromosomal integrations and integrated *o*‐tRNAs; nnAA IgG produced in CF with extract from cells with increasing number of integrated *o*‐tRNAs. E224 is a continuously fermented extract with o‐tRNA transcription from a plasmid. Data in (b) and (c) is from two titer measurements from two independent batches of extract. Error bars show 1 standard deviation. nnAA IgG titers from extracts e299 and e319 are significantly different with *p* < 0.01. CF, cell free; nnAA, nonnatural amino acids

Site‐specific ADC production with CFPS requires co‐translational incorporation of nnAAs (Yin et al., 2017). This requires three additional components in the cell‐free reaction: nnAA with an orthogonal bioconjugation handle, an amber suppressor tRNA not recognized by endogenous *E. coli* synthetases, and an aminoacyl tRNA synthetase capable of specifically recognizing the nnAA and charging it onto the amber suppressor tRNA (Wang et al., 2001). Producing either of the macromolecular components for nnAA incorporation in the extract strain would eliminate a reagent, simplify the reaction, and reduce the costs of producing ADCs with CFPS. First, we wanted to focus on the amber suppressor tRNA because it is the most resource‐intensive reagent. Endogenous tRNA and amber suppressor tRNAs are typically expressed from tRNA operons with a tRNA promoter and terminator (Wang et al., 2001). For our first set of experiments, we wanted to test whether one could transcribe an nnAA tRNA with an alternative promoter during extract fermentation. A small library was created with the natural *proK* tRNA promoter, or strong synthetic promoters including the MTL promoter, PL6 promoter, and a mutant MTL promoter with a 1 bp deletion in the −10 site, see Table S1 for sequences and details, which were used to drive GFP production off a plasmid with the *pheV* terminator. The results from the GFP expression experiment can be seen in Figure 3a. From this data it is clear that this small collection of promoters spans around 1 order of magnitude in transcriptional strength as measured by GFP fluorescence. Novel tRNA transcription cassettes were created with the same terminator and series of promoters. These tRNA transcription cassettes were knocked onto the *araB* locus on the *E. coli* chromosome. Extract was made from the integrant tRNA library members after biomass growth with continuous fermentation. All of these chromosomally integrated tRNA transcription cassettes were very well tolerated, and each of these strains was able to maintain a growth rate (µ) of at least 0.3 h^−1^ in continuous fermentation. The *o*‐tRNA activity in these samples was measured by its potential for amber suppression at position F404TAG in the HC of an anti CD74 IgG in a cell free reaction with the extract as the sole source for *o*‐tRNA (Figure 3b). Here, antibody titer in the CFPS reaction is a surrogate for amber suppression since full length protein can only be transcribed when the nnAA is efficiently incorporated into the growing polypeptide chain at the amber codon. The amber suppression activity in these extract samples correlated well with the GFP levels observed with the off‐plasmid tests of promoter strengths. This means tRNA activity from a given operon is well correlated with the strength of the promoter used to transcribe it. Strain SBDG266 with the MTL promoter, the strongest promoter tested, drives maximal *o*‐tRNA transcription resulting in the most efficient amber suppression and highest antibody titers in CFPS. Figure 3b shows that the IgG titers in strains with a single integrated *o*‐tRNA operon were significantly improved when reactions were supplemented with additional tRNA from the e23 tRNA reagent lysate (Zimmerman et al., 2014). This indicates *o*‐tRNA activity and amber suppression were limiting titers in these extracts. Compared with the single *o*‐tRNA integrants, extract made from strain S224 with tRNA transcription from a multicopy plasmid yielded more efficient amber suppression, likely because more copies of the *o*‐tRNA cassette led to higher levels of tRNA. Previously developed methods exist for integrating multiple copies of genes onto the chromosome to achieve plasmid‐like copy numbers, but these methods suffer from a lack of control over the exact copy number of integrated genes (Tyo et al., 2009). Excess *o*‐tRNA levels had been observed to be deleterious for cell‐free reaction titers, so we were interested in a precise *o*‐tRNA titration. This was achieved by sequentially integrating single *o*‐tRNA expression cassettes in defined locations within the chromosome as single, or tandem tRNA constructs using the MTL promoter to drive transcription. After four more integrations with the cassette containing one or two copies of tRNA, mutants with exact tRNA copy numbers ranging from 1 to 9 were produced. Each of these cell lines was grown in a chemostat then used to prepare extract and used for cell‐free reactions producing aCD74 F404pAMF IgG. From Figure 3c, it can be seen that strains with three or fewer integrated tRNAs resulted in nnAA containing IgG titers which were lower than the control extract, e224, which had tRNA expression driven from a plasmid. For these strains, suboptimal o‐tRNA activity was limiting amber suppression. Strains with 4–6 integrated tRNAs had optimal aCD74 expression and nnAA IgG titers at or above strain S224 with plasmid‐based *o*‐tRNA transcription. Additional copies of tRNA beyond this leads to statistically significant decreases in titer. This may be a result of having too much *o*‐tRNA which has been previously reported to negatively affect the titers of proteins in CFPS (Zimmerman et al., 2014). Strain SBDG299 with four individual integrations of the pAzPhe tRNA was chosen for further work because this *o*‐tRNA configuration maximized titer, amber suppression, and specific growth rate (µ > 0.6 h^−1^) with the fewest chromosomal alterations and *o*‐tRNAs (Figure 3c).

### 3.5 Comparability of continuous extract quality

**Figure 4:**
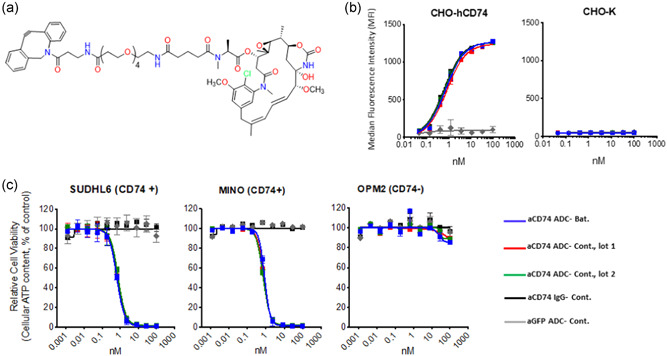
Comparison of antibody–drug conjugates (ADCs) produced in cell free protein synthesis using batch and continuous extract. Extract was made using either a batch fermentation or a continuous fermentation from strain SBDG299. (a) DBCO‐maytansine warhead used to arm aCD74 ADC. (b) ADCs conjugated from mAbs produced with batch extract or two different lots of continuous extract were used for in vitro cell binding with the indicated cell lines. (c) Cell killing of ADCs in (b) with the indicated cells lines. SUDHL6 and MINO cells are CD74 positive and OPM2 cells are CD74 negative. aGFP ADC is an isotype control ADC generated from an IgG whose variable regions target the protein GFP

Strain SBDG299 was capable of stably co‐expressing DsbC, FkpA, and *o*‐tRNA during a multiday continuous fermentation. As a final test, we wanted to assess whether extract made from this strain with continuous fermentation produced ADCs with titers and quality similar to extracts produced in batch fermentation. Extract from SBDG299 was made using either batch or continuous fermentation and then used to produce aCD74 F404pAMF IgG. Table 3 shows that extract generated with continuous fermentation can produce nnAA‐IgG with titers at or above the levels of extract made with batch fermentation. The nnAA‐IgG made in these reactions was purified with protein A affinity chromatography, polished with SEC chromatography, and then conjugated with a cytotoxic maytansine warhead (Figure 4a). The assembly status of the purified IgG was monitored at each step using SEC and the conjugation efficiency was measured with MS. The percentage of fully assembled IgG, partially assembled fragments and aggregate were similar in the proA capture and polished material with both sources of extract.

**Table 3:** Titer, product quality, and conjugation of aCD74 ADC produced in batch and continuous extract

		Batch extract	Continuous extract
CF titer of ProA capture by A_280_		0.45 ± 0.073 g/L^a^, ^b^	0.56 ± 0.033 g/L^a^, ^b^
SEC purity of ProA pool	% Monomer	74%	88%
	% High MW	19%	5%
	% Low MW	7%	8%
SEC purity of polished mAb	% Monomer	98%	97%
	% High MW	0%	0%
	% Low MW	2%	2%
SEC purity of conjugated ADC	% Monomer	96%	96%
	% High MW	2%	2%
	% Low MW	3%	3%
ADC DAR by MALDI‐TOF MS		1.96	1.95

Abbreviations: ADC, antibody–drug conjugate; CF, cell free; DAR, Drug Antibody Ratio; MALDI‐TOF MS, matrix‐assisted laser desorption ionization time‐of‐flight mass spectrometry.

^a^ Titers are listed ± 1 standard deviation.

^b^ Batch and continuous titers are not significantly different with *p* > 0.08.

Each extract produced good quality ADCs with 96% of the final product as fully assembled IgG and DAR > 1.90 (Table 3, Figures S6 and S7). To test the functional comparability of these two different lots of material, they were each used in cell binding and cell killing assays with cells that expressed or lacked the CD74 cell surface marker (Figures 4b,c, and S8). These assays test the bioconjugation potential of the pAMF nnAA and the functional binding activity of the Fab CDRs. The aCD74 IgGs and ADCs made using continuous or batch extract have overlapping cell‐binding activity (Figure 4b). In Figure 4c, all three aCD74 ADCs have expected killing activity with CD74 expressing cells, but not on negative cell lines. The cell‐killing curves for all three lots of aCD74 ADC are indistinguishable indicating that the ADC products made with continuous and batch extract are indistinguishable with in vitro assays.

## 4 DISCUSSION

The manufacture of protein biologics with CFPS would be facilitated by having access to the large volumes of extract enabled with continuous fermentation. This study demonstrates that it is possible to stably grow cells that have been engineered for cell‐free synthesis of nnAA IgGs in a chemostat. For the production of biologics, day‐to‐day consistency is important. We discovered that while it is possible to achieve very high titers of chaperone expression from a plasmid, this system cannot achieve stable expression over the course of a five‐day continuous fermentation. On the other hand, the chromosome is a privileged location for the overexpression of these genes with much‐improved stability. We have demonstrated that it is possible to achieve chaperone co‐expression at levels more than 1 g/L during continuous fermentation over a period of 5 days. This level of chaperone expression is sufficient to support high‐level IgG expression and assembly in CFPS (Groff et al., 2014).

To produce IgGs for making site‐specific ADCs with click chemistry, it is necessary to incorporate nnAAs with biorthogonal reactivity. An amber suppressor tRNA is typically supplied as a separate extract or reagent in CFPS reactions. These reactions potentially have large *o*‐tRNA requirements and the large volumes of this reagent necessary for scaling up can be avoided if the tRNA can also be supplied by the extract. Initially, we showed that the *o*‐tRNA could be expressed of the chromosome during extract fermentation with a strong, synthetic promoter. A single o‐tRNA transcription cassette on the chromosome was not sufficient for maximal amber suppression of cell‐free produced nnAA IgGs. After testing variants with 1–8 copies of integrated o‐tRNA, it appears that four integrations placing 4–6 copies of tRNA at distinct locations throughout the chromosome produce the optimal level of amber suppressor tRNA. By controlling the number and locations of *o*‐tRNA integration, it was possible to achieve amber suppression exceeding extract produced with tRNA transcribed from a plasmid. In addition, this narrow optima for o‐tRNA number would be challenging to achieve with stochastic integration methods. Having both the tRNA and chaperones on the chromosome is also helpful for future strain engineering work. Because there is no plasmid in this strain, additional biochemical factors can be tested with plasmid‐based expression.

Of crucial importance, the synthesis and performance of nnAA IgGs and corresponding ADCs appear identical with cell‐free reactions using either continuous extract or batch extract. SBDG299 extract produced with continuous fermentation leads to cell‐free titers at least as good as extracts made using batch fermentation. The quality of the IgGs produced in terms of fully assembled IgG, aggregate, and partially assembled IgG is very similar with both extract production methods. The conjugation and performance in in vitro cell killing assays for ADCs made with either method are indistinguishable. Continuous fermentation can produce high‐quality extract over extended periods of time with stable co‐expression of three different macromolecular components for nnAA IgG synthesis from the chromosome. Access to large volumes of high‐quality extract will be critical for enabling economical CFPS of ADCs at manufacturing scales.

## CONFLICT OF INTERESTS

The authors declare that there are no conflict of interests.

## AUTHOR CONTRIBUTIONS

Dan Groff, Alex Steiner, Trevor J Hallam, and Gang Yin conceived and designed the experiments. Dan Groff, Nina A. Carlos, Rishard Chen, Jeffrey A. Hanson, Shengwen Liang, Stephanie Armstrong, and Sihong Zhou conducted the experiments. Dan Groff, Nina A. Carlos, Jeffrey A. Hanson, Xiaofan Li, and Gang Yin wrote the manuscript. All authors helped to revise the manuscripts.

## Supporting information

Supporting information.

## Data Availability

All data are available upon request.
