# Supplementary material for: Development of an E. coli strain for cell‐free ADC manufacturing
Source: Biotechnol Bioeng. 2021 Oct 25;119(1):162–75. doi: 10.1002/bit.27961 (PMC9297987; doi:10.1002/bit.27961)
Supplement: Supplementary file 1 — Supporting information. [file BIT-119-162-s001.docx]

Figure S1. Plasmid retention with glutamine auxotrophic selection in continuous fermentation. Samples of inoculum or continuous fermentation broth were diluted into LB and then plated onto LB agar plates with no antibiotics and LB plates with Kan. Percentage of cells retaining plasmid was calculated by dividing number of colonies on Kan by number of colonies on no antibiotic plates. Black bars show the average of three biological replicates shown in white.

Figure S2. Growth of stains for individual auxotrophic systems. Graphs of data shown in Figure [1](#MEP_L_fig1)C for individual *cysE* (left)*, glnA* (middle), and *argA* (right) auxotrophic systems.

Figure S3. ELISA measurements of extract DsbC concentrations during continuous fermentation from samples with chromosomal DsbC overexpression. Extract samples were made at the indicated timepoints and DsbC levels were measured with a coating ELISA using polyclonal DsbC antisera. 108 is a batch extract with DsbC overexpressed from the chromosome.

Figure S4. PAGE analysis of extract chaperone concentrations. 1▒µl of extract samples from the indicated timepoints were analyzed with polyacrylamide gel electrophoresis. FkpA in S161 is expressed from a plasmid and decreases over time. FkpA in S150 is overexpressed from the chromosome and stable during continuous fermentation. Purified DsbC and FkpA were run as standards.

Figure S5. FkpA overexpression from a plasmid leads to culture heterogeneity. S161 Cells were grown continuously in a chemostat. After 5 days, cells were harvested and isolated by plating on LB agar + 50▒µg/ml kanamycin. 8 colonies were picked into terrific broth with 50▒µg/ml kanamycin and grown until an OD600▒=▒2.0. Pellets were lysed by 3 rounds of freeze thawing and soluble protein was analyzed by PAGE. FkpA overexpression was only evident in 2 of 8 clones marked by *. The other 6 clones were resistant to Kan, but didn’t exhibit FkpA overexpression, indicating their FkpA expression cassette had been disrupted.

Figure S6. Analytical SEC chromatogram comparing A) proA elution B) prep-SEC polished C) DBCO-maytansine conjugated aCD74 IgG produced in CFPS reactions utilizing batch (black) and continuous (blue) extract

Figure S7. Reduced MALDI-TOF MS analysis of aCD74 HC conjugated to SC236 produced in CFPS reactions utilizing batch (black) and continuous (blue) extract. Unconjugated HC is shown in red for comparison.

Figure S8. Cell killing activity of free SC236 warhead. Killing curve for ADC is shown in black and for free warhead is shown in green. aCD74 ADC has potency for CD74 positive cell lines 3 orders of magnitude higher than the free drug. Killing is similar for both compounds with CD74 negative cell lines.

Table S1 Constitutive promoter sequences used in this study

| **Name** | **seq uppercase** | **comment** | **source** |
| --- | --- | --- | --- |
| proK | TTAAGGGATTGACGAGGGCGTATCTGCGCAGTAAGATGCGCCCCG | Native E. coli tRNA promoter | Ecocyc.org |
| Pc0 | CTTGACGCTGCGTAAGGTTTTTGTTATAATACACCGCGGG | Medium synthetic promoter | a |
| MTL | ATTATGTCTTGACATGTAGTGAGTGGGCTGGTATAATGCAGCAAG | Strong synthetic promoter | b |
| Mut MTL | ATTATGTCTTGACATGTAGTGAGTGGGCTGGTAAATGCAGCAAG | 1▒bp deletion mutant of MTL pro. | b |
| PL6 | ATTACAACTTGACGTGACATTTTTCCCTGTTATAATGCGCCCCG | Strong synthetic promoter | c |
| CP42 | CATTCGTAAGTTTATTCTTGACACCTGAGATGAGGCGTGATATAATAAATAAGTACTGTT | Medium synthetic promoter | c |
| CP9 | CATTCGTAAGTTTACAACTTGACGGCCCATTGCTTTGGGTTATAATAAATAAGTACTGTT | Strong synthetic promoter | c |

a Groff et al. MAbs. May-Jun 2014;6(3):671-8

b CP25 (http://parts.igem.org/Part:BBa_K1509003), with modified -10 sequence based on (Nucleic Acids Res. 1983 Apr 25; 11(8): 2237–2255).

c synthetic, constitutive promoters designed using strategy from Jensen, P. and Hammer, K. AEM, Jan 1998;64(1):82-87.
